# 2-(Pyrimidin-2-ylsulfan­yl)acetic acid

**DOI:** 10.1107/S1600536809006400

**Published:** 2009-02-28

**Authors:** Jian Xin Pan, Qian Wang Chen

**Affiliations:** a Hefei National Laboratory for Physical Sciences at the Microscale and Department of Materials Science & Engineering, University of Science and Technology of China, Hefei 230026, People’s Republic of China

## Abstract

The mol­ecule of the title compound, C_6_H_6_N_2_O_2_S, lies on a crystallographic mirror plane with the methyl­ene H atoms related by mirror symmetry. In the crystal packing, mol­ecules are linked into layers by inter­molecular O—H⋯N and C—H⋯O hydrogen bonds.

## Related literature

For the coordination chemistry of thio­ether ligands, see: Li & Bu (2008 ▶); Bu *et al.* (2003 ▶); Chen *et al.* (2003 ▶); Demadis & Coucouvanis (1995 ▶); Peng *et al.* (2006 ▶). For bond-length data, see: Allen *et al.* (1987 ▶).
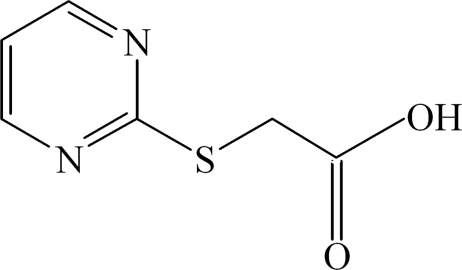


## Experimental

### 

#### Crystal data

C_6_H_6_N_2_O_2_S
*M* *_r_* = 170.19
Orthorhombic, 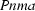

*a* = 14.660 (6) Å
*b* = 6.579 (2) Å
*c* = 7.664 (3) Å
*V* = 739.2 (5) Å^3^
*Z* = 4
Mo *K*α radiation
μ = 0.38 mm^−1^
*T* = 153 K
0.22 × 0.20 × 0.07 mm

#### Data collection

Bruker P4 diffractometer
Absorption correction: multi-scan (*SADABS*; Sheldrick, 1996 ▶) *T*_min_ = 0.920, *T*_max_ = 0.974
5392 measured reflections
911 independent reflections
828 reflections with *I* > 2σ(*I*)
*R*_int_ = 0.024

#### Refinement

*R*[*F* ^2^ > 2σ(*F* ^2^)] = 0.032
*wR*(*F* ^2^) = 0.086
*S* = 1.07
911 reflections
70 parameters
H atoms treated by a mixture of independent and constrained refinement
Δρ_max_ = 0.22 e Å^−3^
Δρ_min_ = −0.20 e Å^−3^

### 

Data collection: *SMART* (Siemens, 1996 ▶); cell refinement: *SAINT* (Siemens, 1994 ▶); data reduction: *SAINT*; program(s) used to solve structure: *SHELXS97* (Sheldrick, 2008 ▶); program(s) used to refine structure: *SHELXL97* (Sheldrick, 2008 ▶); molecular graphics: *SHELXTL* (Sheldrick, 2008 ▶); software used to prepare material for publication: *SHELXTL*.

## Supplementary Material

Crystal structure: contains datablocks I, global. DOI: 10.1107/S1600536809006400/rz2292sup1.cif

Structure factors: contains datablocks I. DOI: 10.1107/S1600536809006400/rz2292Isup2.hkl

Additional supplementary materials:  crystallographic information; 3D view; checkCIF report

## Figures and Tables

**Table 1:** Hydrogen-bond geometry (Å, °)

*D*—H⋯*A*	*D*—H	H⋯*A*	*D*⋯*A*	*D*—H⋯*A*
O2—H1⋯N2^i^	0.89 (3)	1.78 (3)	2.663 (2)	178 (3)
C6—H6⋯O1^ii^	0.93	2.44	3.266 (3)	148
C5—H5⋯O2^iii^	0.93	2.47	3.403 (3)	178

Symmetry codes: (i) 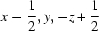; (ii) 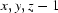; (iii) 
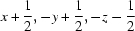
.

**Fig. 1.**
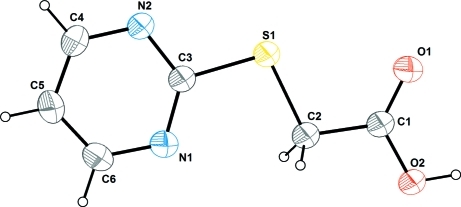
The molecular structure of the title compound, with the atom-numbering scheme. Displacement ellipsoids are drawn at the 50% probability level.

## supplementary crystallographic information

### Comment

Great efforts have been focused on the rational design and synthesis of metal-organic coordination architectures by using flexible bridging ligands due to their flexibility and conformational freedoms, which offer the possibility for the construction of unprecedented frameworks (Li & Bu, 2008). Recently, flexible thioethers have been well established ligands in coordination and metallosupramolecular chemistry because of their rich structural information (Bu *et al.*, 2003; Chen *et al.*, 2003; Demadis & Coucouvanis, 1995; Peng *et al.*, 2006). In the process of preparing metal-organic coordination architectures, single-crystals of the title compound were obtained unexpectedly.

The molecular structure and the atom-numbering scheme of the title compound are shown in Fig. 1. The molecule lies on a mirror plane, with the methylene H atoms related by mirror symmetry. All bond lengths (Allen *et al.*, 1987) and angles show normal value. In the crystal packing, molecules are linked into layers perpendicular to the *b* axis by intermolecular O—H···N and C—H···O hydrogen bonds (Table 1).

### Experimental

A mixture of Co(Ac)_2_.6H_2_O (0.142 g, 0.50 mmol), (2-Pyrimidylthio)acetic acid (0.035 g, 0.20 mmol) and sodium azide (0.032 g, 0.50 mmol) in H_2_O (10 ml) was stirred for 1 h, then filtered, and the filtrate was kept at room temperature. Single crystals of the title compound were obtained by slow evaporation of the solvent after a few days.

### Refinement

Hydrogen atoms bound to C atoms were positioned geometrically with C—H = 0.93-0.97 Å, and constrained to ride on their parent atoms, with *U*_iso_(H) = 1.2*U*_eq_(C). The H atom bound to O was freely refined.

### Crystal data

**Table:**

C_6_H_6_N_2_O_2_S	*F*(000) = 352
*M**_r_* = 170.19	*D*_x_ = 1.529 Mg m^−^^3^
Orthorhombic, *P**n**m**a*	Mo *K*α radiation, λ = 0.71073 Å
Hall symbol: -P 2ac 2n	Cell parameters from 2019 reflections
*a* = 14.660 (6) Å	θ = 4.3–27.5°
*b* = 6.579 (2) Å	µ = 0.38 mm^−^^1^
*c* = 7.664 (3) Å	*T* = 153 K
*V* = 739.2 (5) Å^3^	Prism, colorless
*Z* = 4	0.22 × 0.20 × 0.07 mm

### Data collection

**Table:**

Bruker P4 diffractometer	911 independent reflections
Radiation source: fine-focus sealed tube	828 reflections with *I* > 2σ(*I*)
graphite	*R*_int_ = 0.024
ω scans	θ_max_ = 27.5°, θ_min_ = 4.3°
Absorption correction: multi-scan (*SADABS*; Sheldrick, 1996)	*h* = −18→18
*T*_min_ = 0.920, *T*_max_ = 0.974	*k* = −8→7
5392 measured reflections	*l* = −9→9

### Refinement

**Table:**

Refinement on *F*^2^	Primary atom site location: structure-invariant direct methods
Least-squares matrix: full	Secondary atom site location: difference Fourier map
*R*[*F*^2^ > 2σ(*F*^2^)] = 0.032	Hydrogen site location: inferred from neighbouring sites
*wR*(*F*^2^) = 0.086	H atoms treated by a mixture of independent and constrained refinement
*S* = 1.07	*w* = 1/[σ^2^(*F*_o_^2^) + (0.0435*P*)^2^ + 0.2357*P*] where *P* = (*F*_o_^2^ + 2*F*_c_^2^)/3
911 reflections	(Δ/σ)_max_ < 0.001
70 parameters	Δρ_max_ = 0.22 e Å^−^^3^
0 restraints	Δρ_min_ = −0.20 e Å^−^^3^

### Special details

**Table:**

Geometry. All e.s.d.'s (except the e.s.d. in the dihedral angle between two l.s. planes) are estimated using the full covariance matrix. The cell e.s.d.'s are taken into account individually in the estimation of e.s.d.'s in distances, angles and torsion angles; correlations between e.s.d.'s in cell parameters are only used when they are defined by crystal symmetry. An approximate (isotropic) treatment of cell e.s.d.'s is used for estimating e.s.d.'s involving l.s. planes.
Refinement. Refinement of *F*^2^ against ALL reflections. The weighted *R*-factor *wR* and goodness of fit *S* are based on *F*^2^, conventional *R*-factors *R* are based on *F*, with *F* set to zero for negative *F*^2^. The threshold expression of *F*^2^ > σ(*F*^2^) is used only for calculating *R*-factors(gt) *etc*. and is not relevant to the choice of reflections for refinement. *R*-factors based on *F*^2^ are statistically about twice as large as those based on *F*, and *R*- factors based on ALL data will be even larger.

### Fractional atomic coordinates and isotropic or equivalent isotropic displacement parameters (Å^2^)

**Table:**

	*x*	*y*	*z*	*U*_iso_*/*U*_eq_	Occ. (<1)
S1	0.36018 (3)	0.2500	0.16539 (6)	0.0429 (2)	
O2	0.09853 (9)	0.2500	0.24621 (18)	0.0421 (4)	
H1	0.067 (2)	0.2500	0.345 (4)	0.088 (10)*	
O1	0.21943 (9)	0.2500	0.42130 (18)	0.0516 (4)	
N2	0.50091 (10)	0.2500	−0.0392 (2)	0.0373 (4)	
C2	0.24089 (12)	0.2500	0.1111 (3)	0.0415 (5)	
H2A	0.2262	0.3696	0.0426	0.050*	0.50
H2B	0.2262	0.1304	0.0426	0.050*	0.50
C3	0.40934 (12)	0.2500	−0.0428 (2)	0.0334 (4)	
C4	0.54355 (13)	0.2500	−0.1933 (3)	0.0408 (5)	
H4	0.6070	0.2500	−0.1960	0.049*	
C1	0.18678 (12)	0.2500	0.2774 (2)	0.0349 (4)	
N1	0.35745 (10)	0.2500	−0.1845 (2)	0.0395 (4)	
C6	0.40261 (14)	0.2500	−0.3361 (2)	0.0437 (5)	
H6	0.3690	0.2500	−0.4390	0.052*	
C5	0.49614 (14)	0.2500	−0.3477 (3)	0.0446 (5)	
H5	0.5259	0.2500	−0.4549	0.054*	

### Atomic displacement parameters (Å^2^)

**Table:**

	*U*^11^	*U*^22^	*U*^33^	*U*^12^	*U*^13^	*U*^23^
S1	0.0244 (3)	0.0774 (4)	0.0270 (3)	0.000	−0.00018 (16)	0.000
O2	0.0221 (6)	0.0738 (10)	0.0304 (7)	0.000	−0.0008 (5)	0.000
O1	0.0293 (7)	0.0959 (12)	0.0298 (7)	0.000	−0.0031 (5)	0.000
N2	0.0246 (7)	0.0571 (10)	0.0304 (8)	0.000	0.0002 (6)	0.000
C2	0.0251 (8)	0.0698 (14)	0.0298 (9)	0.000	−0.0004 (7)	0.000
C3	0.0262 (8)	0.0448 (10)	0.0292 (8)	0.000	−0.0004 (7)	0.000
C4	0.0284 (9)	0.0549 (12)	0.0391 (10)	0.000	0.0054 (7)	0.000
C1	0.0254 (8)	0.0481 (11)	0.0311 (9)	0.000	−0.0012 (7)	0.000
N1	0.0293 (8)	0.0593 (11)	0.0299 (8)	0.000	−0.0015 (6)	0.000
C6	0.0403 (11)	0.0632 (13)	0.0277 (9)	0.000	−0.0041 (8)	0.000
C5	0.0416 (10)	0.0624 (14)	0.0299 (9)	0.000	0.0075 (8)	0.000

### Geometric parameters (Å, °)

**Table:**

S1—C3	1.7507 (19)	C2—H2B	0.9700
S1—C2	1.7976 (19)	C3—N1	1.326 (2)
O2—C1	1.316 (2)	C4—C5	1.372 (3)
O2—H1	0.89 (3)	C4—H4	0.9300
O1—C1	1.202 (2)	N1—C6	1.337 (2)
N2—C4	1.336 (2)	C6—C5	1.374 (3)
N2—C3	1.343 (2)	C6—H6	0.9300
C2—C1	1.501 (3)	C5—H5	0.9300
C2—H2A	0.9700		
			
C3—S1—C2	100.92 (9)	N2—C4—H4	119.2
C1—O2—H1	111 (2)	C5—C4—H4	119.2
C4—N2—C3	116.72 (16)	O1—C1—O2	123.91 (17)
C1—C2—S1	108.51 (13)	O1—C1—C2	124.65 (16)
C1—C2—H2A	110.0	O2—C1—C2	111.44 (16)
S1—C2—H2A	110.0	C3—N1—C6	115.33 (17)
C1—C2—H2B	110.0	N1—C6—C5	123.38 (18)
S1—C2—H2B	110.0	N1—C6—H6	118.3
H2A—C2—H2B	108.4	C5—C6—H6	118.3
N1—C3—N2	126.17 (17)	C4—C5—C6	116.72 (18)
N1—C3—S1	120.70 (14)	C4—C5—H5	121.6
N2—C3—S1	113.13 (13)	C6—C5—H5	121.6
N2—C4—C5	121.68 (18)		

### Hydrogen-bond geometry (Å, °)

**Table:**

*D*—H···*A*	*D*—H	H···*A*	*D*···*A*	*D*—H···*A*
O2—H1···N2^i^	0.89 (3)	1.78 (3)	2.663 (2)	178 (3)
C6—H6···O1^ii^	0.93	2.44	3.266 (3)	148
C5—H5···O2^iii^	0.93	2.47	3.403 (3)	178

Symmetry codes: (i) *x*−1/2, *y*, −*z*+1/2; (ii) *x*, *y*, *z*−1; (iii) *x*+1/2, −*y*+1/2, −*z*−1/2.
